# Updated and standardized genome-scale reconstruction of *Mycobacterium tuberculosis* H37Rv, iEK1011, simulates flux states indicative of physiological conditions

**DOI:** 10.1186/s12918-018-0557-y

**Published:** 2018-03-02

**Authors:** Erol S. Kavvas, Yara Seif, James T. Yurkovich, Charles Norsigian, Saugat Poudel, William W. Greenwald, Sankha Ghatak, Bernhard O. Palsson, Jonathan M. Monk

**Affiliations:** 1 Department of Bioengineering, University of California, San Diego, La Jolla, CA USA; 2 Bioinformatics and Systems Biology Program, University of California, San Diego, La Jolla, USA; 3 Department of Pediatrics, University of California, San Diego, La Jolla, CA USA; 4 Novo Nordisk Foundation Center for Biosustainability, Technical University of Denmark, Kemitorvet, Building 220, 2800 Kongens Lyngby, Denmark

**Keywords:** *M. tuberculosis*, Genome-scale reconstruction, Environmental condition, Antibiotic resistance

## Abstract

**Background:**

The efficacy of antibiotics against *M. tuberculosis* has been shown to be influenced by experimental media conditions. Investigations of *M. tuberculosis* growth in physiological conditions have described an environment that is different from common in vitro media. Thus, elucidating the interplay between available nutrient sources and antibiotic efficacy has clear medical relevance. While genome-scale reconstructions of *M. tuberculosis* have enabled the ability to interrogate media differences for the past 10 years, recent reconstructions have diverged from each other without standardization. A unified reconstruction of *M. tuberculosis* H37Rv would elucidate the impact of different nutrient conditions on antibiotic efficacy and provide new insights for therapeutic intervention.

**Results:**

We present a new genome-scale model of *M. tuberculosis* H37Rv, named iEK1011, that unifies and updates previous *M. tuberculosis* H37Rv genome-scale reconstructions. We functionally assess iEK1011 against previous models and show that the model increases correct gene essentiality predictions on two different experimental datasets by 6% (53% to 60%) and 18% (60% to 71%), respectively. We compared simulations between in vitro and approximated in vivo media conditions to examine the predictive capabilities of iEK1011. The simulated differences recapitulated literature defined characteristics in the rewiring of TCA metabolism including succinate secretion, gluconeogenesis, and activation of both the glyoxylate shunt and the methylcitrate cycle. To assist efforts to elucidate mechanisms of antibiotic resistance development, we curated 16 metabolic genes related to antimicrobial resistance and approximated evolutionary drivers of resistance. Comparing simulations of these antibiotic resistance features between in vivo and in vitro media highlighted condition-dependent differences that may influence the efficacy of antibiotics.

**Conclusions:**

iEK1011 provides a computational knowledge base for exploring the impact of different environmental conditions on the metabolic state of *M. tuberculosis* H37Rv. As more experimental data and knowledge of *M. tuberculosis* H37Rv become available, a unified and standardized *M. tuberculosis* model will prove to be a valuable resource to the research community studying the systems biology of *M. tuberculosis*.

**Electronic supplementary material:**

The online version of this article (10.1186/s12918-018-0557-y) contains supplementary material, which is available to authorized users.

## Background

The success of *M. tuberculosis* as a pathogen has been partially attributed to its unique metabolic capabilities. The metabolic network of *M. tuberculosis* has evolved to withstand and navigate the harsh environment imposed by the alveolar macrophage. Most bacteria cannot thrive in this hypoxic, acidic and nutrient-limited condition, yet it is in this harsh environment that *M. tuberculosis* encounters and evolves resistance to antibiotics. Elucidating the robust properties of metabolism that enable *M. tuberculosis* pathogenicity and drug resistance evolution has become a key area of research.

Recent studies have demonstrated that the choice of experimental media conditions plays an important role in understanding physiologically-relevant phenotypes of *M. tuberculosis* [1]. Commonly used experimental media conditions such as Middlebrook 7H9 are known to be much different from the physiological environment. For example, despite extensive research describing fatty acids as a key carbon source within the macrophage environment, most studies forgo the inclusion of fatty acids in the media, opting instead for glucose or glycerol [2]. Perhaps it is no surprise then that differences between the in vitro and in vivo environments have been shown to affect antibiotic screening results [3–11]. In particular, it has been shown that hypoxic or nutrient limited conditions alter the metabolism of *M. tuberculosis* to a nonreplicating, drug-resistant state [5–7]. Specific mechanism-changing effects between in vitro and in vivo conditions have been shown to occur for many antibiotics [9, 10].

While it is understood that differences in experimental media conditions lead to phenotypic variations, dissecting a mechanistic understanding of these different phenotypes remains challenging. Genome-scale models (GEMs) of metabolisms have emerged as powerful tools to computationally probe the effect of media composition on a cell’s phenotype [12]. For the past 10 years, GEMs have provided a mechanistic basis for exploring the metabolic capabilities of *M. tuberculosis* on the systems-level. GEMs of *M. tuberculosis* have helped interrogate a variety of biological phenomena, from understanding the transcriptional regulatory network [13] to elucidating metabolic interactions between *M. tuberculosis* and the alveolar macrophage [14].

While new *M. tuberculosis* H37Rv GEMs have enabled novel insights, they have been constructed from different base models resulting in divergent representations of the metabolic network. For example, gene-protein-reaction rules (GPRs) (i.e., the Boolean relationship between a gene, or set of genes, and the corresponding reaction(s)) differ within reactions shared amongst models (e.g., The GPR of Rv0904c differs between iOSDD and iSM810). In addition to variation in network topology, divergent GEMs have a variety of identifiers used for metabolite and reaction names, making them difficult to compare and build from (e.g., “R#” reaction identifier nomenclature used in most models built primarily off of GSMN-TB). While such differences may seem insignificant, the presence of multiple divergent *M. tuberculosis* H37Rv reconstructions hinders progress and may result in future wasted efforts [15].

Here, we present iEK1011, a new GEM of *M. tuberculosis* H37Rv that unifies, standardizes, and updates previous divergent GEMs of this model organism. We assess the performance of iEK1011 to that of previous GEMs through gene essentiality prediction on two different datasets. iEK1011 is further characterized by performing simulations that examine the model’s predictions in physiological conditions and interrogate differences between in vitro and in vivo media conditions. Finally, in order to provide a comprehensive platform for elucidating antibiotic resistance (AMR), we integrate knowledge derived from experimental literature into iEK1011.

## Results

### Workflow for updating, unifying, and standardizing previous reconstructions of M. tuberculosis

In order to ensure a comprehensive unification, we first gathered and compared available reconstructions of *M. tuberculosis* H37Rv. Since the first two *M. tuberculosis* H37Rv reconstructions released in 2007 [16, 17], a total of 9 reconstructions have been built (Table 1). Most models were largely based off of either iNJ661 [16] or GSMN-TB [17]. Specifically, out of the most recent *M. tuberculosis* reconstructions, sMtb [18], iSM810 [13] and gal2015 [19] were primarily built from GSMN-TB while iOSDD [20] was built from iNJ661.

**Table 1:** Summary of existing genome-scale models of M. tuberculosis. iAB-AMØ-1410-Mt-661 has over 2000 genes because it combines an updated version of iNJ661 with a macrophage model

Model	Year	Genes	Reactions	Metabolites	Reference
iNJ661	2007	661	1025	826	[16]
GSMN-TB	2007	726	856	645	[17]
MMF-RmwBo	2009	776	1108	???	[56]
HQMTB	2009	686	607	734	[57]
iNJ661v	2010	663	1049	838	[58]
iAB-AMØ-1410-Mt-661	2010	2071	4489	3400	[14]
MergedTBmodel	2012	917	1400	1017	[59]
GSMN-TB1.1	2013	759	876	667	[60]
iOSDD890	2014	890	1152	961	[20]
sMtb	2014	915	1192	929	[18]
gal2015	2015	760	965	754	[19]
iSM810	2015	810	938	723	[13]
iNJ661mu	2016	672	1057	846	[61]
iEK1011	2017	1011	1228	998	This study

The model provided by *Garay* et al. was given the name of gal2015 because it is unnamed in the original publication

**Fig. 1:**
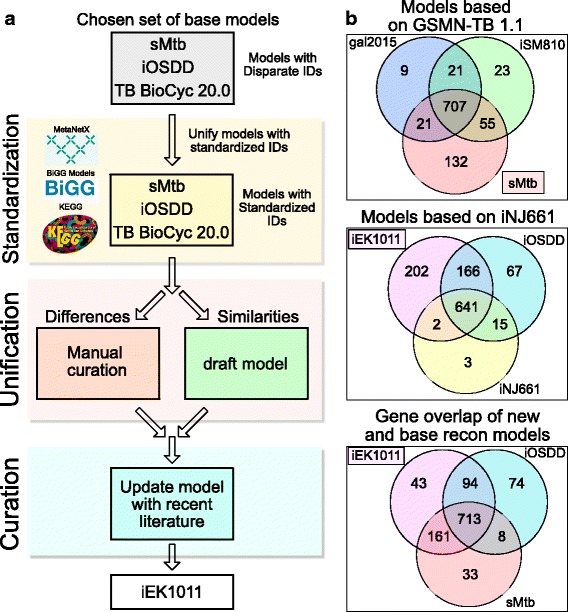
**a** Workflow of reconstruction process. A draft GEM model was built from the TB BioCyc 20.0 database and mapped to BIGGs IDs along with sMtb and iOSDD (see Additional file 2). The models were then unified by first joining the similarities between them, followed by manual curation of model differences literature and database validation. **b** Overlap of genes across different model sets. The model that covers most of the models within the particular set is enclosed by a box

Using a variety of both quantitative and qualitative criteria (e.g., standardized identifiers, gene essentiality predictions, mass balanced reactions; see Methods), iOSDD and sMtb were chosen as the base reconstructions for the unification process (Fig. 1a). The recently developed *M. tuberculosis* H37Rv BioCyc Database [21] provided an additional reconstruction resource to supplement the standardized draft model. The reconstruction process was performed following a clear workflow (Fig. 1a): the base models were mapped to standardized BiGG identifiers [22], joined into a draft model of shared reactions and unified by assessing model disagreements. The resulting unified draft model was then expanded through manual curation of new biochemical knowledge. Thus, the reconstruction process was iterative and involved constant re-evaluation of model content (see Methods).

The resulting unified and updated reconstruction of *M. tuberculosis* H37Rv, named iEK1011, contains 1011 genes, 1228 reactions, and 998 metabolites. iEK1011 encapsulates the majority of genes in the previous models based on either iNJ661 or GSMN-TB (Fig. 1b). iEK1011 accounts for 96% of sMtb genes (874 of 915 genes) and 91% of iOSDD genes (807 of 890). A total of 151 unique genes from iOSDD, iNJ661, gal2015, iSM810, and sMtb were not accounted for in iEK1011 (see Additional file 1) either due to insufficient evidence necessary to resolve major inconsistencies across models or lack of confidence in gene annotation.

In addition to unifying previous reconstructions, iEK1011 incorporated 39 new genes absent from previous models. In particular, sulfur metabolism was updated by adding the *cysO*-dependent biosynthesis of L-cysteine, which connects molybdenum metabolism with sulfur metabolism through the use of *moeZ* in both pathways [23]. New pathways and reactions include heme uptake [24], tuberculosinol biosynthesis [25], ergothioneine biosynthesis [26, 27], and mycobilin biosynthesis [28]. The resulting unified reconstruction of *M. tuberculosis*, iEK1011, provides a biochemically-derived knowledge-base that can be functionally assessed computationally.

### Functional assessment of iEK1011

iEK1011 was converted to a mathematical model to examine the functional capabilities of the improved reconstruction and to quantitatively compare it with previous reconstructions. The primary tool for evaluating genome-scale reconstructions of *M. tuberculosis* H37Rv has been in silico gene essentiality testing. Therefore, we used gene essentiality as a metric for evaluating and comparing the performance of iEK1011. Gene essentiality predictions across previous *M. tuberculosis* H37Rv reconstructions were determined using the same data and quantitative score used in evaluating the predictive ability of iSM810 [13]. In addition to the commonly used gene essentiality dataset by *Griffin* et al. [29], a recent gene essentiality dataset by *DeJesus* et al. [30] was also utilized in our model comparisons. The primary differentiating feature between the datasets was the media condition used to generate them (see Additional file 1). Using these gene-essentiality datasets, we evaluated and compared the ability of five models (iNJ661, iOSDD, sMtb, iSM810, and iEK1011) to predict gene essentiality.

**Fig. 2:**
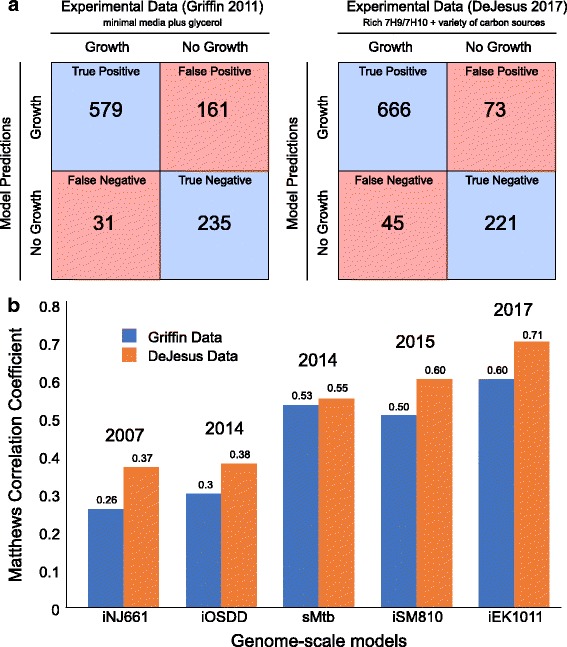
Gene Essentiality Prediction Comparisons. **a** Model-predicted gene essentiality results compared to both the *Griffin* et al. and *deJesus* et al. essentiality experimental datasets. **b** Gene essentiality performance using the Matthews Correlation Coefficient. iSM810 and sMtb, which were both built off of GSMN-TB 1.1, significantly outperform iNJ661 and iOSDD. iEK1011 outperforms all models on both gene essentiality datasets

When using the Griffin dataset, we found that iEK1011 increases the prediction of true positives (i.e., the model correctly predicts growth for the gene knockout when the gene is annotated as non-essential) by 23% (579) (Fig. 2a) over sMtb (470), which had the largest number of true positives amongst the previous models. iEK1011 gene essentiality predictions decrease the number of false negatives (i.e., the model incorrectly predicts no growth for the gene knockout when the gene is annotated as non-essential) by 11.4% (31) (Fig. 2a) over iSM810 (35), which had the least number of false negatives amongst the previous models.

With respect to the more recent DeJesus essentiality dataset, iEK1011 increases the number of true positives by 24% (666) (Fig. 2a) over sMtb (538), and iEK1011 increases the number of true negatives by 11% (221) (Fig. 2a) over sMtb (199). iEK1011 decreases the number of false positives by 14% (73) over sMtb (83), and increases the number of false negatives by one (45) over iSM810 (44). The increase in one more false positive over iSM810 is due to having 9 genes tested in the false negative category that are not contained in iSM810. Moreover, relating specific groups, such as false negatives or true positives, against other models with a different number of genes may not correctly represent the changes due to significant differences in class sizes.

In order to account for the variations in class sizes amongst models, we calculated the Matthews Correlation Coefficient (MCC) for each model’s prediction on both gene essentiality datasets (Fig. 2b). iEK1011 scores the highest on both datasets with an MCC of 0.60 and 0.71 on the Griffin and DeJesus dataset, respectively (Fig. 2b) (see Additional file 1). These iEK1011 MCC values are a 6% and 18% increase over the previous best model MCC’s of sMtb and iSM810 on the Griffin and DeJesus dataset, respectively.

Although the DeJesus essentiality dataset is more recent than the Griffin dataset by 6 years, the media condition used in determining essentiality on the DeJesus dataset was not as well defined because it utilized oleic-albumin-dextrose-catalase (OADC) in middlebrook 7H10/7H9 media supplemented with a variety of carbon sources [30]. The contents of OADC are not well defined primarily because of albumin, which may supplement amino acids to *M. tuberculosis*. The extent of OADC’s impact remains unknown, which ultimately hinders the ability to rigorously define the inputs for GEMs, which are crucial components of COBRA methods [31]. Conversely, the media used in Griffin was well defined as minimal media supplemented with glycerol [29]. Therefore, the increase in MCC by 6% over sMtb on the Griffin essentiality data should be evaluated with more confidence than the significantly higher percent increase in MCC over all models on the DeJesus dataset. Thus, the gene essentiality results presented above demonstrate improved predictive capability of iEK1011 over previous *M. tuberculosis* GEMs.

### iEK1011 qualitatively recapitulates flux states indicative of physiologically relevant media conditions

While the gene essentiality predictions are a useful metric to evaluate model quality, we prioritized the model’s ability to recapitulate *M. tuberculosis* behavior described in the literature. Specifically, an emphasis was placed on central carbon metabolism given its distinctive usage in *M. tuberculosis* and recent emergence as an unexpected research frontier [32]. In addition, we focused on *M. tuberculosis* studies involving conditions relevant to pathogenicity [1]. Therefore, we compared simulations between two conditions relevant to the purpose of this study: Lowenstein-Jensen media, representing in vitro drug testing conditions; and an in vivo nutrient condition approximated from the literature that attempts to replicate the pathogenic state. We used Flux Variability Analysis (FVA) [33] and randomized sampling [34] to characterize and compare the fluxes between the two media conditions.

**Fig. 3:**
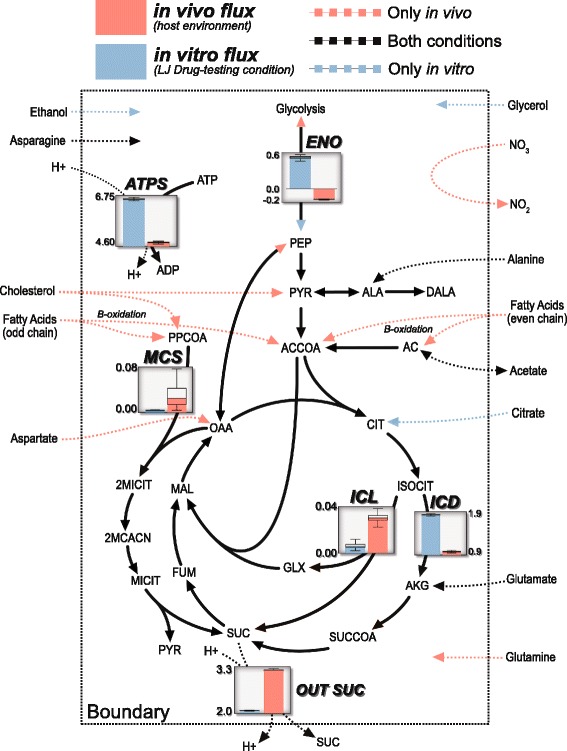
Metabolic map of flux differences through central carbon metabolism in iEK1011 between approximate in vitro and in vivo conditions. The media conditions are represented by nutrients outside of the dotted boundary line. Box plots graphically depict flux differences in the sampled solution spaces between in vivo and in vitro media conditions

Taking advantage of recent studies investigating nitrogen metabolism within the context of *M. tuberculosis* pathogenicity [35–38], we set the in vivo nitrogen sources to be composed of nitrate, aspartate, asparagine, glutamate, urea, and glutamine (Fig. 3**,** see Additional file 1). Under hypoxic in vivo conditions, iEK1011 predicts use of nitrate in a respiratory role as opposed to a nitrogen source where it is taken in and reduced to nitrite by *narG*, and then exported out of the cell, a finding consistent with previous experiments [37]. The chosen in vivo carbon sources include fatty acids (both even and odd chain), cholesterol, CO_2_, and Alanine. Fatty acids were chosen as the primary source of carbon in vivo due to the vast amount of literature evidence supporting the claim that *M. tuberculosis* uses host-derived fatty acids [2, 39, 40]. iEK1011 catabolizes fatty acids through beta-oxidation, which generates acetate (even chain fatty acid catabolism), propionyl-CoA (odd chain fatty acid catabolism) and acetyl-CoA (Fig. 3). Although CO_2_ was incorporated due to evidence showing it being fixated by *M. tuberculosis* in an approximated in vivo environment [38], iEK1011 was not predicted to fixate CO_2_ due to a net gluconeogenic flux through phosphoenolpyruvate carboxykinase - a simulation result also found in *Beste* et al. [38]. Alanine was included as a nutrient due to evidence describing it to be in abundant quantities within the alveolar macrophage and being imported from the macrophage [38].

The differences in flux state simulations predicted by iEK1011 between the two conditions recapitulate key behavior described in the literature. Specifically, in the approximated in vivo condition involving hypoxia and growth on fatty acids, model-predicted flux decreases through TCA with an accompanying increase in succinate secretion (Fig. 3). iEK1011 predicts the secretion of succinate to allow optimal growth in these conditions because it removes an intracellular proton, allowing for membrane potential related reactions such as oxidative phosphorylation to proceed. This mechanism has been previously described to be specific and essential in *M. tuberculosis* hypoxia adaptation [41]. Thus, iEK1011 can recapitulate known physiological phenomena using stoichiometry alone.

In addition to succinate secretion, iEK1011 simulates the activation of both the glyoxylate shunt and the methylcitrate cycle in response to both hypoxia and growth on fatty acids [41, 42]. Although the median flux values are low (Fig. 3) (based on markov chain monte carlo sampling of the solution space [34]), FVA simulations show maximum flux values through methylcitrate cycle and glyoxylate shunt to have a threefold and twofold increase in in vivo media conditions relative to in vitro conditions, respectively **(**see Additional file 1). Furthermore, the metabolic model does not account for the toxic effect of glyoxylate and propionate which has been shown to necessitate flux through glyoxylate shunt and methylcitrate cycle. While iEK1011 simulations do not account for characteristics like toxicity, the examples outlined above show that iEK1011 is capable of qualitatively recapitulating key phenomena uncovered in recent years.

### iEK1011 as a computational knowledge base for interrogating features of antibiotic resistance

We have shown that iEK1011 is a valuable source of computational inquiry through gene essentiality predictions and its ability to recapitulate phenomena described in the literature. In addition to providing a computational platform, GEMs are fundamentally a knowledge-base that are capable of contextualizing a variety of concepts that extend beyond the genome-scale metabolic network [31]. Taking advantage of this ability to incorporate abstractions, we translate knowledge derived from experimental investigations of antibiotic resistance (AMR) evolution into a format that can be integrated into GEMs.

**Fig. 4:**
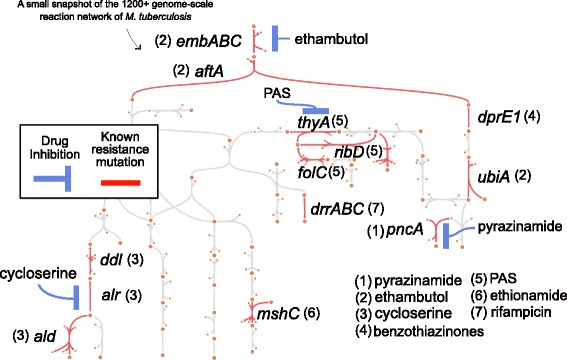
Escher map of arabinogalactan-peptidoglycan complex biosynthesis with known resistance-conferring genes mapped. The gene-antibiotic relation is indicated by the number placed proximal to the gene. The mechanistic effect by the antibiotic is indicated by the blue line. No blue line is shown for mutations in which the gene-antibiotic relation remains unclear (i.g., *mshC*, *drrBC*), Escher-usable maps were built for multiple subsystems in iEK1011 (see Additional file 4)

Using the extensive literature on the mechanism of AMR evolution in *M. tuberculosis*, we curated a relational table between antibiotics, genes, and metabolic reactions for eight different antibiotics (Table 2). The genes associated with a particular antibiotic are those known to be central to AMR evolution (i.e., mutations in the genes that code for the reactions often confer resistance to specific drugs). Displaying AMR genes on a metabolic map of iEK1011 portrays relationships that would be difficult to comprehend without a GEM (Fig. 4). Notably, we found that the close topological relationships between para-aminosalicylic acid, ethambutol, D-cycloserine, and pyrazinamide may hint at pleiotropic effects (i.e., mutations that affect multiple phenotypes) of resistance conferring mutations on the efficacy of different antibiotics.

**Table 2:** Table of antibiotics and the associated genes whose mutations confer antibiotic resistance

Drug	Gene	iEK1011 reaction	Reference
Ethambutol	*embABC*	EMB	[62]
	*ubiA*	DCPT	[43]
	*aftA*	AFTA	[43]
D-cycloserine	*alr*	ALAR	[63]
	*ddl*	ALAALAR	[64]
	*ald*	ALAD_L, GXRA	[44]
Isoniazid	*katG*	CAT	[65]
	*inhA*	FAS	[66]
	*fabG1*	MYCSacp56/58/50	[67]
Benzothiazinones	*dprE1*	DCPE	[68]
PAS	*thyA*	TMDS	[51]
	*ribD*	FOLR2, ASPRAUR, DHPPDA2	[51]
	*folC*	DHFS, THFGLUS	[51]
Pyrazinamide	*pncA*	NNAM	[69]
Ethionamide	*mshC*	CIGAMS	[52]
Rifampicin	*drrABC*	PDIMAT, PPDIMAT	[70]

In order to incorporate specific antibiotic pressures into iEK1011, we evaluated each antibiotic and associated a biochemical objective function that approximates the evolutionary drivers of selection (Table 3). In the case of ethambutol, it has been shown that flux-increasing mutations in *ubiA* confer resistance by increasing the production of decaprenylphosphoryl-b-D-arabinose (DPA), which outcompetes ethambutol for *embB* binding spots [43]. Therefore, in a GEM, the evolutionary pressure imposed by ethambutol can be approximated as a metabolic objective where the production of DPA is maximized (Table 3). A total of four antibiotics were associated with approximated objective functions representing evolutionary forces (see Methods for further reasoning of the choice of objective function).

**Table 3:** List of objective functions related to the evolutionary drivers of antibiotic resistance

Drug	Objective	Reaction in iEK1011	Reference
Ethambutol	MAX DPA production	decda__tb_c → □	[43]
PAS	MAX Tetrahydrofolate production	thf_c → □	[51]
D-cycloserine	MAX L-Alanine Production	ala__L_c → □	[44]
Ethionamide	MIN mycothiol production	msh_c → □	[52]

The abbreviations are as follows: *PAS* para-aminosalicylic acid, *MAX* maximize, *MIN* minimize

Taking advantage of the translation of antibiotic features to formats amenable by iEK1011, we simulated the evolutionary pressures induced by antibiotics and calculated the maximum and minimum fluxes for the AMR-associated reactions in both in vivo and in vitro conditions through FVA (see Additional file 1).

**Fig. 5:**
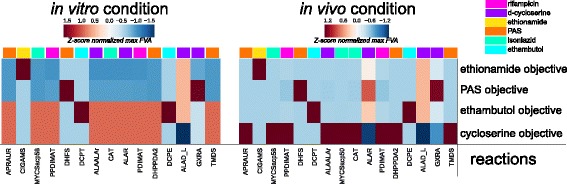
Heatmaps of maximum FVA values for for a matrix representing FVA values for the curated AMR reactions across simulations of different drug-specific objective functions (see Table 2 for curated list of AMR genes and their associated iEK1011 reactions, see Table 3 for drug-specific objectives)

There were few differences in relative flux for a specific drug objective between these conditions. However, those that were uncovered highlighted potential impacts of environmental/media composition differences. In particular, we see major differences in the fluxes that correspond to optimizing the approximated ethambutol-induced evolutionary pressure (see Fig. 5). Furthermore, this ethambutol flux is correlated with fluxes induced by the approximated d-cycloserine objective. Closer inspection of the uptake differences driving these differential flux states points to L-alanine as a key environmental influence. In particular, the differential fluxes within the cases of ethambutol resistance-conferring genes *ubiA* (DCPT) and *embB* (EMB), as well as d-cycloserine resistance-conferring genes of *alr* (ALAR), *ald* (ALAD_L), and *ddlA* (ALAALAr), exemplify the differential effect of environmental L-alanine availability. Notably, L-alanine has been shown to be an important substrate in the macrophage environment (Beste 2013). While L-alanine and other amino acids may be available in LJ drug-testing media due to utilization of egg base or bovine serum, our analysis only accounted for metabolites that were explicitly stated in defined quantities within the media conditions. With respect to the efficacy of antibiotics, these results suggest that d-cycloserine and ethambutol may be less effective in vivo due to increased availability of L-alanine, which is a key precursor reaction catalyzed by AMR genes targeted by d-cycloserine and ethambutol, whereas in vitro conditions may increase susceptibility to ethambutol. In both cases, the significant decrease in model-predicted maximum ALAD_L (*ald*) flux is in line with studies describing the deleterious mutations in *ald* that confer resistance to D-cycloserine [44]. Altogether, iEK1011 provides a knowledge base for relating antibiotic resistance features through genome-scale metabolic network analysis.

## Discussion

The divergence of *M. tuberculosis* H37Rv reconstructions has created an unnecessary obstacle in contextualizing the increasing growth of biochemical data for this troublesome pathogen. In order to address experimental insights to pathogenic conditions and alleviate roadblocks for future reconstruction efforts of *M. tuberculosis* H37Rv, we built a unified and updated GEM of *M. tuberculosis*, iEK1011. We tested the predictive potential of iEK1011 by comparing gene essentiality predictions with previous models and showed that iEK1011 outperforms previous models. We further assessed the predictive capabilities of iEK1011 by comparing simulated flux states between in vitro drug testing and approximate in vivo media conditions. Comparisons recapitulate specific phenomena indicative of biochemical flux states seen in physiological conditions. We incorporated antibiotic resistance knowledge in iEK1011, which enabled a network-based perspective of multi-antibiotic resistance evolution.

iEK1011 unified previous *M. tuberculosis* H37Rv reconstructions and encompassed the majority of genes within the two divergent groups of reconstructions. Additionally, iEK1011 incorporates new pathways including the incorporation of ergothioneine biosynthesis. This addition will aid a quantitative elucidation of the relationships between sulfur metabolism, bioenergetic homeostasis, and redox balance [27]. As a unified, standardized, and updated model, iEK1011 provides a base for future models of *M. tuberculosis* H37Rv.

Functional assessment of previous *M. tuberculosis* H37Rv reconstructions through gene essentiality predictions showed that iEK1011 achieves a higher MCC than previous models on two different datasets. While the two datasets were crucial in both assessing and driving iEK1011 reconstruction, experimental gene essentiality datasets derived from physiologically-relevant conditions are warranted for understanding the human-restricted lifestyle of *M. tuberculosis*.

Using iEK1011, we qualitatively determined differences in biochemical states between in vitro and approximated in vivo conditions. We showed that iEK1011 successfully recapitulates specific phenomena described in physiologically-relevant studies of *M. tuberculosis*. Future reconstruction efforts may target iEK1011’s lack of predicted CO_2_ fixation [38] and account for compartmentalized co-metabolism of multiple substrates [45, 46]. iEK1011 may provide a base for future host-pathogen integrated reconstructions that leverage valuable experimental data.

An integrated knowledge-base of genome-scale metabolism and antibiotic resistance components may enable new perspectives for understanding and combating *M. tuberculosis* H37Rv. We translated experimental knowledge of AMR genes and specific adaptation mechanisms to formats amenable to iEK1011. Comparing simulations of these AMR features between in vitro and in vivo conditions emphasized the potential impact of hypoxia and L-alanine availability on the pressures induced by antibiotics. Future constraint-based analysis of *M. tuberculosis* AMR may leverage new experimental approaches, such as those that have analyzed changes in essentiality under antibiotic exposure [47].

Taken together, iEK1011 is a new, comprehensive and predictive constraint-based model of *M. tuberculosis* H37Rv. In this study, we computationally demonstrate that in vivo nutrient sources absent from in vitro media significantly alter the flux state of central carbon metabolism. As experimental insights to *M. tuberculosis* pathogenicity and antibiotic resistance continue to grow, this GEM will provide a foundation to connect disparate data types and knowledge.

## Methods

### Reconstruction of iEK1011

#### Choosing a base reconstruction

A variety of both quantitative and qualitative criteria was considered in determining which model would provide the base for the new model. The determining criterion included the amount of curated data, extent of previous model unification, gene essentiality predictions, standardized identifiers, cross-references to databases, and quality of physical representations, such as the use of an extracellular compartment and mass balanced reactions. Based on this criterion, the reconstruction of iEK1011 was based on the unification of iOSDD, sMtb, and portions of the *M. tuberculosis* H37Rv BioCyc Database [21] (Fig. 1a).

With regards to the selection criterion, sMtb was chosen as the primary base model. Notably, sMtb performed the best amongst the previous models in gene essentiality predictions (Fig. 2b). In addition, sMtb included metabolite formulas, an extracellular compartment, and cross-references to databases. Both iSM810 and gal2015, which were both built off of GSMN-TB 1.1, lacked standardized identifiers (i.e., reactions identifiers were arbitrarily named R1, R2, etc.), metabolite formulas, and an extracellular compartment (i.e. inputs into the model could be utilized without being transported across the membrane). The lack of chemical formulas disables the assessment of mass conservation, which is a defining feature of constraint-based modeling. Furthermore, an extracellular compartment is key in distinguishing between what goes into the media and what is being transported across the membrane. While iOSDD performed well in categories related to component descriptions*,* it was based on iNJ661 and thus had a lower gene essentiality score, as previously shown [13]. Despite the low gene essentiality score, we utilized iOSDD as a representative for the models based on iNJ661. In this study, we show through gene essentiality predictions that the integration of iOSDD with sMtb results in a 6% increase in gene essentiality predictions.

The reconstruction process was straightforward (Fig. 1a). The base models were first algorithmically mapped to standardized BiGG identifiers (see Additional file 2) [22]. Identifiers that could not be mapped by the algorithm were manually assigned identifiers that follow the BiGGs format. Importantly, BiGGs was chosen as the standardization platform due to being a centralized repository for high-quality models. Once a standardized basis for identifiers was established, a draft reconstruction was built from the set of reactions shared across the standardized models. The differences between reactions across the models were manually assessed through literature references and added to the draft reconstruction. Once the models were unified into the draft reconstruction, manual curation of new biochemical knowledge was incorporated in the reconstruction. The reconstruction process was iterative and involved constant re-evaluation of model components. iEK1011 is provided at the BiGG website [22] initialized with m7H10 media as well as in JSON format with five different media initializations media as well as in JSON format with five different media initializations (see Additional file 3).

#### Updating the reconstruction

The model was updated with newly characterized metabolic processes, standardized identifications, and mass balanced reactions. In addition, detailed and designable metabolic maps of *M. tuberculosis* metabolism were manually built and provided in the supplement (see Additional file 4) in order to help in silico simulation and reconstruction efforts as well as provide access to systems biology research for non-computational biologists. Specifics on using the escher maps are described in the section titled “Escher Flux Maps”.

Before any updating took place, sMtb identifiers for metabolites and reactions were mapped to standardized identifiers in the BiGG Models database [22]. In addition to sMtb*,* the BioCyc *M. tuberculosis* H37Rv version 20.0 database was approximately converted to a cobra model - standardizing it first to BiGGs IDs, then MetaNetx, and then BioCyc identifiers if no BiGGs or Metanetx reference mapping was available. When an sMtb component had no equivalent BiGGs identification, a new identifier was created that followed the BiGG’s nomenclature. The updated reconstruction utilizes data from Tuberculist, 2016 TB BioCyc database, and manually curated literature sources. New pathways and major GPR updates include Tuberculosinol biosynthesis, oxidized GTP and dGTP detoxification, Heme uptake and degradation, GlgE pathway update, glucosylglycerate biosynthesis I, included essential genes Rv3805c and Rv2673 in MAP complex biosynthesis, and others (see Additional file 1). In addition to incorporating updates from the new BioCyc database, we re-curated pathways that had inconsistencies across divergent models.

#### Description of GAM and NGAM parameters

Our model includes both growth-associated (GAM) and non-growth (NGAM) associated ATP maintenance parameters. NGAM quantifies the energy required by Mtb to maintain itself in a given environment while GAM quantifies growth energy requirements not accounted for in the metabolic model. For iEK1011, the GAM was chosen to be 60 mmol gDw^− 1^, which was the same as the GAM used in previous *M. tuberculosis* H37Rv reconstructions of iNJ661, iAB-AMØ-1410-Mt-661, and iOSDD. For comparison, the GAM used for sMtb—a model built from the GSMN-TB line of reconstructions—was 57 mmol gDw^− 1^. For the NGAM, iEK1011 uses a value of 3.15 mmol gDw^− 1^ h^− 1^, which was taken from the *E. coli* model [48]. For comparison, the NGAM used in sMtb was set to 0.1 mmol gDw^− 1^ h^− 1^, while the NGAM used in iSM810 was 1 mmol gDw^− 1^ h^− 1^. We are not aware of any datasets available for *M. tuberculosis* that enables a rigorous evaluation of the NGAM parameter, such as those used for *E. coli* [49] (i.e., quantitative substrate uptake rates for different substrates).

In order to assess the sensitivity of our chosen NGAM, we recomputed the gene essentiality using an NGAM value of 1.0 and 0.01. With respect to our previous NGAM of 3.15, the NGAMs of 1.0 and 0.1 result in very similar values (see Additional file 5: Table S1). We hope that future experimental efforts will enable a better parameterization of GAM and NGAM in genome-scale reconstructions of *M. tuberculosis*.

### Future directions

#### Inconsistencies between fatty acid metabolic pathways amongst models

There is a major difference in fatty acid metabolism between the previous models. ACP metabolites, holo enzymes, FAS metabolites, and other metabolites within the fatty acid metabolic pathways make relating and joining these pathways very challenging. iEK1011 primarily uses the form of the fatty acid pathways described in iOSDD, which significantly departs from the GSMN-TB based models and sMtb. We computationally profiled switches between forms taken in iOSDD and those in sMtb and found minor differences in simulations. The key feature maintained across divergent models was that the odd and even chain fatty acids described in the media went into the methylcitrate cycle and TCA, respectively.

In order to help future efforts in coming up with a consensus fatty acid metabolic pathway for a *M. tuberculosis* reconstruction, we have additionally included models named “sMtb_mapped_model.json” and “biocyc_sMtb_joined.json” (see Additional file 4) that have the fatty acid pathways of sMtb and BioCyc mapped to BiGGs identifiers.

#### Lumped reactions that violate mass conservation

We have tabulated out the lumped reactions in iEK1011 that violate mass conservation (Additional file 5: Table S2). These reactions were taken from previous models and are lumped in order to join parallel pathways. Although these reactions are kept in iEK1011, we hope that future reconstructions of *M. tuberculosis* H37Rv will work towards having all reactions mass balanced. Despite these disadvantages, we believe that iEK1011 is a step in the correct direction and allows for convergent and additive knowledge.

#### Blocked reactions in iEK1011

Out of the 1228 reactions in iEK1011, 157 are blocked (i.e., reactions that can’t carry flux even with all exchange reactions open). We have provided a list of these 157 blocked reactions (see Additional file 1). The subsystem with the highest frequency of blocked reactions is “Cofactor and Prosthetic Group Biosynthesis” (34 reactions), followed by “Exchange” (21 reactions) and “Transport” (10 reactions). Thus, future efforts should target the blocked reactions in the subsystem of “Cofactor and Prosthetic Group Biosynthesis” in order to optimize the reconstruction process.

#### Gene essentiality predictions

Gene essentiality predictions were determined using the same data and quantitative score used in evaluating the predictive ability of iSM810 [13]. The gene essentiality dataset was acquired from *Griffin* et al. [29]. If the Griffin essentiality confidence score was less than 0.1, the gene was determined to be essential. A growth cutoff of 20% of optimal growth was chosen to determine whether the in silico knockout was essential or not (i.e. if it was less than 20% of optimal growth, the gene was determined to be essential).

In addition to the Griffin gene essentiality dataset, we also evaluated the performance of the models in using a recent gene essentiality dataset acquired from *DeJesus* et al. [30]. A cutoff of 20% was used for the DeJesus dataset for the gene annotations of GD (growth defect), ES (essential), and ESD (essential domain). If growth was above 20% of optimal growth, the gene was said to be NE (non-essential) and GA (growth advantage). The Matthews Correlation Coefficient was used to score the quality of each model’s prediction, given by the following equation:

$$ MCC=\frac{TP\ast TN- FP\ast FN}{\sqrt{\left( TP+ FP\right)\left( TP+ FN\right)\left( TN+ FP\right)\left( TN+ FN\right)}} $$

where TP (True Positive) represents the event where the model correctly simulates growth when a gene is nonessential. TN (True Negative) represents the event where the model correctly simulates no-growth when a gene is essential. FP (False Positive) represents the event where the model simulates no growth with the gene knockout when the gene is in fact non-essential. FN (False Negative) represents the event where the model simulates growth when the gene is in fact essential. While the *Griffin* et al. essentiality dataset is older, we utilized it due to having a more defined media conditions and was previous used in previous *M. tuberculosis* H37Rv reconstruction studies. The default objective function (“biomass”) was used across all models. Differences in MCC values between this study and that in *Ma* et al. [13] are due to differences in growth cutoff thresholds and media conditions. Despite inconsistencies, the values remained similar and did not change the resulting 6% increase in gene essentiality by iEK1011. Specifics regarding which genes are falsely predicted in all models, which pathways iEK1011 fails, which pathways iEK1011 performs correct predictions are provided (see iEK1011_supplementary.xlsx sheets titled “All models incorrect”, “iEK Wrong and sMtb Correct”, and “iEK Correct and sMtb Wrong”).

We further verified that the increase in accuracy for the iEK model is due to model curation as opposed to the addition of new non-essential genes by comparing only the gene essentiality predictions of the genes shared across all models. In particular, only 472 genes are shared across all the models. Using this set of genes for all model gene essentiality predictions, we find that iEK1011 still increases accuracy over other models (see Additional file 5: Table S4). Specifically, iEK1011 achieves 57% MCC on the Griffin dataset while sMtb achieves 50% MCC. On the de Jesus dataset, iEK1011 achieves 66% MCC while iSM810 achieves 55%. In the first case, iEK1011 has + 8 TP, + 9 TN, − 9 FP, and − 8 FN over sMtb. In the second case, iEK1011 has + 2 TP, + 25 TN, − 25 FP, and − 2 FN over iSM810. These results show that the increase in accuracy is due to model curation. This analysis has been appended to the provided ipython notebook “reproducible_modeling.ipynb”, and the results are attached in the supplementary excel file “iEK1011_supplementary.xlsx” under the sheets labeled “Shared essentiality predictions the Griffin et al. dataset” and “Shared essentiality predictions the deJesus dataset”, under sheets 9 and 10, respectively.

#### Flux variability analysis and sampling of in vitro and in vivo conditions

All constraint-based simulations of iEK1011 were done using the python constraint-based modeling package, COBRApy [50]. While the linear program is guaranteed to find the global optimum, the flux state solution to this optimization problem may not be unique, leading to the alternate optimal flux states. To account for this, we ran Flux Variability Analysis (FVA) in both the Lowenstein-Jensen media and approximated in vivo conditions using the “biomass” objective function (see Additional file 1). FVA gives the maximum and minimum amount of flux a reaction can take on. In addition to FVA, we sampled the solution space of iEK1011 on both media conditions using markov-chain monte-carlo sampling (MCMC) package available in cobrapy.

Furthermore, for both FVA and MCMC sampling, we allowed for solutions within 95% of the optimal value. The growth rate for both simulations were approximately the same to allow for a meaningful quantitative flux value comparisons.

#### Approximation of literature-derived evolutionary forces of antibiotic-resistance evolution

A more in depth reasoning for the choice of objective function is described below for each antibiotic.

##### Ethambutol

It has been shown that flux-increasing mutations in *ubiA* confer resistance to ethambutol by increasing the production of decaprenylphosphoryl-b-D-arabinose (DPA), which outcompetes ethambutol for *embB* bindings spots [43]. Therefore, we approximate the evolutionary force of adaptation as maximizing the production of DPA.

##### D-cycloserine

Analogous to the mechanism of ethambutol, it has been shown that loss-of-function mutations in *ald* confer resistance to the d-cycloserine by increasing the pool of Alanine (i.e. *ald* no longer converts Alanine to pyruvate), thereby competitively inhibiting d-cycloserine [44].

##### Para-aminosalicylic acid (PAS)

Mutations in *folC*, *ribD*, and *thyA* have been shown to confer resistance to PAS [51]. It was suggested that *thyA* mutations are selected in order to decrease the utilization of folates. In addition, it was suggested that the up-regulation of *ribD* occurs as an alternative when *dfrA* is inhibited.

##### Ethionamide

It has been shown that mycothiol biosynthesis is essential for ethionamide susceptibility [52]. We approximate ethionamide resistance is minimizing the production of mycothiol. It is worth noting that the objective defined for ethionamide is a much looser approximation than the other objectives defined before.

#### Comparison of FVA across different drug objective simulations

For both in vivo and in vitro media conditions, we simulated each of the drug objectives and compared the maximum and minimum fluxes of the reactions catalyzed by the curated antibiotic resistance genes (see Table 3 main text). The maximum and minimum fluxes for each reaction were determined by FVA (described above) allowing for solutions within 95% of optimum. Furthermore, iEK1011 was constrained to produce at least 20% of biomass growth (i.e., the lower bound of the “biomass” reaction was set to frac*max_biomass_growth, where max_biomass_growth is the optimum value of iEK1011 when maximizing biomass on either in vivo or in vitro conditions), and frac is the percentage of biomass to maintain while optimizing the other objective functions. All simulations can be performed using the provided IPython notebook (see Additional file 6). 

#### Escher flux maps

In order to help future in silico simulations and reconstruction efforts of *M. tuberculosis*, we have provided detailed, manually constructed escher maps of central carbon metabolism, nitrogen metabolism, sulfur metabolism, and arabinogalactan-peptidoglycan complex biosynthesis [53] (see Additional file 4). Escher maps are designable metabolic maps that allow for the integration of different data types with a genome-scale metabolic model. Furthermore, the provided Escher maps will hopefully enable non-computational biologists to explore the complex network of *M. tuberculosis* in an intuitive manner; simply download the json map file(s), go to http://escher.github.io/, and load in the json map. If the model is loaded in as well, you will be able to modify reactions on the map or build your own. In addition, you can view the flux states generated by iEK1011 on the Escher map.

## Additional files

Additional file 1: Sheet 1: Reactions in iEK1011 and details of reactions. Sheet 2: Metabolites in iEK1011 and details of metabolites. Sheet 3: Genes present in previous models but not accounted for in iEK1011. Sheet 4: Gene Essentiality Predictions with the Griffin et al. dataset. Sheet 5: Gene Essentiality Predictions with the deJesus dataset. Sheet 6: Description of media conditions used in study. Sheet 7: Maximum FVA values for AMR genes across different objectives on in vivo media. Sheet 8: Maximum FVA values for AMR genes across different objectives on in vitro media. Sheet 9: “Shared” Essentiality Predictions with the Griffin et al. dataset. Sheet 10: “Shared” Essentiality Predictions with the deJesus et al. dataset. Sheet 11: List of blocked reactions in iEK1011. Sheet 12: “All models incorrect” - List of genes that were incorrectly predicted across all models and their corresponding iEK1011 subsystems. Sheet 13: “iEK Wrong and sMtb Correct” - List of incorrect iEK1011 predictions that were correctly predicted by sMtb. Sheet 13: “iEK Correct and sMtb Wrong” - List of correct iEK1011 predictions that were incorrectly predicted by sMtb. (XLSX 258 kb)

Additional file 2: A conversion of the sMtb model to BiGGs identifiers (JSON 476 kb)

Additional file 3: iEK1011 models: Genome-scale models of iEK1011 in json format initialized with different media conditions. iEK1011_griffinEssen_media.json - Used for essentiality testing on *Griffin* et al. dataset. iEK1011_deJesusEssen_media.json - Used for essentiality testing on *deJesus* et al. dataset. iEK1011_m7H10_media.json - iEK1011 initialized with Middlebrook 7H10 media. iEK1011_drugTesting_media.json - Used for simulating on Lowenstein-Jensen media. iEK1011_inVivo_media.json - Used for simulating on approximated physiological media. (ZIP 310 kb)

Additional file 4: Escher Maps: Contains four escher maps of *M. tuberculosis* metabolic subsystems. Central_carbon.json. Arabinogalactan_peptidoglycan_complex.json. Nitrogen.json. Sulfur_and_folate.json (ZIP 236 kb)

Additional file 5: **Table S1.** Table describing changes in gene essentiality predictions according to changes in GAM and NGAM values that were utilized across different genome-scale reconstructions of *M. tuberculosis* .**Table S2.** List of reactions in iEK1011 that violate the law of mass conservation. **Table S3.** Examples of false negatives computed by iEK1011 on the DeJesus et al. gene essentiality dataset that are not within the iSM810 model, and reasoning for its inclusion [54, 55]. **Table S4.** Gene essentiality predictions using the shared set of 472 genes. (DOCX 17 kb)

Additional file 6: An ipython notebook that runs the simulations described in this study. (IPYNB 637 kb)

## Abbreviations

AMR: Antimicrobial resistance
DPA: Decaprenylphosphoryl-b-D-arabinose
FVA: Flux variability analysis
MCC: Matthews correlation coefficient
PAS: Para-aminosalicylic acid
