# Supplementary material for: Updated and standardized genome-scale reconstruction of Mycobacterium tuberculosis H37Rv, iEK1011, simulates flux states indicative of physiological conditions
Source: BMC Syst Biol. 2018 Mar 2;12:25. doi: 10.1186/s12918-018-0557-y (PMC5834885; doi:10.1186/s12918-018-0557-y)
Supplement: Supplementary file 5 — Table S1. Table describing changes in gene essentiality predictions according to changes in GAM and NGAM values that were utilized across different genome-scale reconstructions of M. tuberculosis .Table S2. List of reactions in iEK1011 that violate the law of mass conservation. Table S3. Examples of false negatives computed by iEK1011 on the DeJesus et al. gene essentiality dataset that are not within the iSM810 model, and reasoning for its inclusion [54, 55]. Table S4. Gene essentiality predictions using the shared set of 472 genes. (DOCX 17 kb) [file 12918_2018_557_MOESM5_ESM.docx]

**Table S1**: Table describing changes in gene essentiality predictions according to changes in GAM and NGAM values that were utilized across different genome-scale reconstructions of *M. tuberculosis*.

|  | iEK1011 NGAM | iSM810 NGAM | sMtb NGAM |
| --- | --- | --- | --- |
| NGAM | 3.15 (iEK1011) | 1.00 (iSM810) | 0.10 (sMTb) |
| Griffin essentiality MCC | 0.60 | 0.60 | 0.59 |
| DeJesus essentiality MCC | 0.71 | 0.70 | 0.70 |

**Table S2**: List of reactions in iEK1011 that violate the law of mass conservation.

| **Unbalanced Reactions** | **iEK1011 reaction string** |
| --- | --- |
| **TAG** | 10.0 12dgr_TB_c + arachcoa_c + hexccoa_c + nodcoa_c + pentdcoa_c + 2.0 pmtcoa_c + 3.0 stcoa_c --> 10.0 coa_c + 10.0 tag_TB_c |
| **LIPY** | 10.0 h2o_c + 9.0 tag_TB_c --> 9.0 12dgr_TB_c + arach_c + 2.0 hdca_c + 2.0 hexc_c + 4.0 ocdca_c |
| **VIUB** | atp_c + fe3_e + h2o_c + 0.0005 mcbts_c + 0.0005 mcbtt_c --> adp_c + fe3_c + pi_c |
| **MBTA1** | acac_c + h_c + n6hlys_c + n6hlysmal_c + nadh_c + salc_c + thr__L_c --> 6.0 h2o_c + mcbtt_c + nad_c |
| **MMM2r** | 0.001 aqcobal_c + succoa_c <-- mmcoa__R_c |
| **MME** | mmcoa__R_c <-- 0.001 aqcobal_c + mmcoa__S_c |
| **NMO** | etha_c + fmnh2_c + o2_c --> acald_c + fmn_c + no2_c |

**Table S3**: Examples of false negatives computed by iEK1011 on the DeJesus et al. gene essentiality dataset that are not within the iSM810 model, and reasoning for its inclusion.

| IEK1011 FNs | In iSM810? | Reasoning |
| --- | --- | --- |
| Rv0505c | No but in others | Evidence of gene. Could be regulatory. |
| Rv2895c (viuB) | No but in others | Iron uptake… but described not to be necessary for iron uptake *in vitro*... |
| Rv1739c | No but in others | Sulfate uptake |
| Rv1159 | No but in others | Strong evidence of annotation [68]. Oddly left out of iSM810 when the other non-FNs were included in phosphatidylinositol mannosides (PIMS). |
| Rv3807c | No, only in iOSDD | Putative evidence [69] . Noted in TB Biocyc Database 21.1 The fact that it is not essential may mean that the annotation is incorrect. |
| Rv3825c | No, but in sMtb. | Strong evidence [70] |

**Table S4**: Gene essentiality predictions using the shared set of 472 genes.

|  | Griffin Essentiality Data | | | | | deJesus Essentiality Data | | | | |
| --- | --- | --- | --- | --- | --- | --- | --- | --- | --- | --- |
|  | MCC | TP | TN | FP | FN | MCC | TP | TN | FP | FN |
| iEK1011 | 0.57 | 160 | 206 | 86 | 21 | 0.66 | 195 | 195 | 49 | 32 |
| sMtb | 0.50 | 152 | 197 | 95 | 29 | 0.53 | 180 | 179 | 65 | 47 |
| iSM810 | 0.45 | 154 | 178 | 114 | 27 | 0.55 | 193 | 170 | 74 | 34 |
| iOSDD | 0.24 | 148 | 122 | 170 | 33 | 0.30 | 186 | 114 | 130 | 41 |
| iNJ661 | 0.22 | 141 | 129 | 163 | 33 | 0.27 | 177 | 119 | 125 | 50 |
